# Comparison of gender identification using exfoliated cells obtained from toothbrush and miswak: A longitudinal study

**DOI:** 10.1097/MD.0000000000038401

**Published:** 2024-06-07

**Authors:** Abdullah Alqarni, Shaik Mohamed Shamsudeen, Master Luqman Mannakandath, Shaik Mohammed Asif, Saeed Alassiri, Khalil Ibrahim Assiri

**Affiliations:** a Department of Dental Diagnostic Science & Oral Biology King Khalid University, Abha, Saudi Arabia.

**Keywords:** exfoliated oral cells, gender identification, miswak, polymerase chain reaction, toothbrush

## Abstract

Gender identification plays a pivotal role in forensic medicine. Among the various methods used for gender identification, deoxyribose nucleic acid (DNA) based methods are considered accurate. Exfoliated oral mucosal cells that are harvested from oral hygiene aids can be potentially used for gender identification using real-time polymerase chain rection (PCR). The aim of the present longitudinal study is to assess and compare the efficacy of toothbrush and miswak as potential tools to harvest exfoliated cells for gender identification. Forty healthy volunteers were recruited and asked to clean their teeth using new toothbrush and fresh miswak each day for 4 days. Toothbrush and miswak used by the participants were subjected to DNA analysis immediately, 1st, 2nd and 6th month. The absorbance of DNA samples were quantified and gender identification was done by amplification of sex determining gene-Sex determining region Y gene (SRY) and ALT1 genes using real-time PCR. The number of correct and positive identification for samples at various time points were tabulated and subjected to statistical analysis. Post hoc power analysis showed that the study had a power of 93%. Correct and positive gender identification was observed for the samples (100%) obtained using miswak, for tooth brush it reduced to 95%, 80%, and 35% at the end of 1^st^, 2^nd,^ and 6^th^ month. The differences seen at the end of 2^nd^ month and 6^th^ month were statistically significant. Miswak is a better tool to harvest exfoliated cells for gender identification when compared to a toothbrush. Hence, miswak can serve as a potential tool in forensic medicine for DNA extraction and subsequently victim identification.

## 1. Introduction

Identification of human remains following crimes, accidents, natural disasters and war falls under the purview of forensic medicine. In situations like this, human identification is crucial in helping the police and legal system, providing comfort to impacted parties, and looking into the nature of the disaster or accident and those involved.^[1–3]^ The first step in human identification, popularly referred to by forensic anthropologists as the “big 4,” is identifying the age, gender, race, and stature.^[4]^ Forensic analysis including analysis of palatal ridge pattern, dermatoglyphic patterns, cheiloscopy have been adequately documented.

Advances in molecular biology have facilitated the identification of victims even when the ante-mortem record is unavailable. The human genome sequencing and the comparatively economic polymerase chain rection (PCR) method of amplifying deoxyribose nucleic acid (DNA) have made DNA analysis a routine operation in basic sciences and clinical medicine.^[5]^ Using PCR, severely deteriorated material can be used to harvest DNA from a postmortem specimen which can subsequently be processed and compared to a close biological relative.^[2]^ In the absence of biological relations, the identification is made possible by comparison with known biological samples of the deceased, such as hair from a hairbrush, epithelial cells from a toothbrush, or a biopsy sample^[2,3]^ Thus, there is a strong need to identify various inanimate objects used by the victim from which genetic material can be harvested for potential matching and identification. In forensic odontology, apart from oral mucosal cells, pulp tissue, odontoblastic processes and cellular cementum, exfoliated cells from the oral cavity have been studied as potential source for DNA isolation.^[6,7]^ Saliva-stained stamps, cigarette butts, toothbrushes, oral prosthetics are some of the inanimate objects that could be potential sources for harvesting DNA.^[2]^

Chewing sticks, which were introduced by Babylonians approximately 7000 years ago, were widely in use before the advent of modern-day toothbrushes.^[8]^ In 1987, World Health Organization promoted the use of chewing sticks for maintenance of oral hygiene in regions which do not have access to toothbrushes. Miswak (*Salvadora persica*), a popular chewing stick used for almost 1000 years by Muslims, is becoming increasingly common as it is considered an obligatory religious practice.^[9–11]^ Miswak can serve as a direct and an affordable tool to harvest the exfoliated oral mucosal cells which can be used subsequently for DNA extraction and person identification. A recently conducted study comparted the purity and yield of DNA material obtained from miswak and toothbrush.^[3]^

Considering this as a prelude, the current study sought to compare the efficacy of miswak and toothbrush as being potential tools to harvest exfoliated oral mucosal cells for subsequent identification of gender from the DNA at the different time intervals. The research hypothesis was that toothbrushes and miswak did not differ in their efficacy to harvest exfoliated oral mucosal cells.

## 2. Materials and methods

### 2.1. Ethical registration details

The present study was conducted at Department of Diagnostic Dental Science after obtaining ethical clearance from Institutional Ethical Committee (Register number: SRC/ETH/2018- 19/143).

### 2.2. Eligibility criteria and recruitment of study participants

The purpose of the study was explained to all out-patients visiting the hospital and voluntary sampling was opted. The volunteers of 18 to 60 years of age were included in the study. Those who were immunocompromised, pregnant, undertaking any medications due to systemic illness and having any type of oral mucosal lesions were excluded. Based on the above-mentioned inclusion and exclusion criteria 40 volunteers (20 males and 20 females) were chosen and recruited into this study. Informed consent was obtained from all the participants.

### 2.3. Details of the experiment

All the volunteers were provided with 4 medium sized toothbrushes and 4 miswaks. All of the volunteers were instructed to use a toothbrush in the morning and miswak in the evening every day for 4 days. On each day, they were instructed to use a fresh toothbrush and a fresh miswak. The principal investigator collected the used toothbrush and miswak from each participant every day. The collected toothbrush and miswak samples were transported to a laboratory in a sterile pouch. The initial analysis was carried out immediately after the collection of the first set of toothbrushes and miswaks. The next set of toothbrushes and miswaks obtained from the participants at Day 2, Day 3, and Day 4 were stored in the laboratory at room temperature and analyses were carried out at the end of 1st month, 2nd month, and 6th month, respectively.

### 2.4. Sample preparation

The head of each toothbrush and miswak was removed individually using a heated scalpel. The removed portions were immersed in TE-buffer and were subjected to incubation through vigorous shaking in cold water for 3 hours. By doing centrifugation at 3000 rpm for 10 minutes, the cell debris were pelleted.^[12]^

### 2.5. DNA extraction

Aidar and Line methods were used to extract the Genomic DNA from buccal epithelial cells of tooth brush and miswak samples.^[8]^ The tubes were centrifuged to pellet the buccal cells. Proteinase K was added for cell lysis. The supernatant obtained was poured off and the pellet was washed with ethanol for a careful decantation. Following centrifugation, the tubes were reversed and air-dried on an absorbent paper. The DNA was re-suspended in 100 liters of TE-buffer and was stored at −20ºC.^[3]^

### 2.6. DNA quantification

The absorbance of each DNA sample was quantified using a Nano drop spectrophotometer at 260 and 280 nm.^[6]^

### 2.7. Sex determination through RT-PCR

The sex determination was done by amplification of sex determining region on Y chromosomes (SRY) and X chromosomes (ALT1 gene) respectively using real-time polymerase chain reaction (RT-PCR).

The sequences of oligonucleotide primers were:

5’-CAT GAA CGC ATT CAT CGT GTG GTC-3’; and 5’-CTG CGG GAA GCA AAC TGC AAT TCT T-3’ for SRY

5’-CCC TGA TGA AGA ACT TGT ATC TC-3’; and 5’-GAA ATT ACA CAC ATA GGT GGC ACT3’ for ATL1

Each 15 µL PCR reaction comprised of 25 to 50 ng of the retrieved DNA from the sample, 1.5 µL of 10X buffer II (Perkin Elmer), 1 µL of 25 mmol dNTP, 1.5 mM MgCl2, 1 pmol each of SRY and ATL1 primers, and 0.25 unit Taq DNA polymerase. A thermal cycler at 94°C (2 minutes) was used to perform initial DNA denaturation. This was followed by DNA denaturation for 35 cycles at 94°C for 15 seconds. 65°C (20 seconds) was used for primer annealing and 72°C (20 seconds) for primer extension. The cycle final extension was done at 72°C for 10 minutes.^[16]^ The presence of respective target genes (SRY for males and ALT for females) were automatically detected by Real Plex Master Cycler.^[6]^

If a sample detected its respective gender target gene after PCR amplification, it was marked as positive. The number of samples showing such positive identification was noted for both miswaks and toothbrushes separately. This was done at 4 time points such as immediately after obtaining the sample, after 1 month, after 2 months and after 6 months. The number of toothbrush samples identified with correct gender were compared with that of miswak at all these 4 time points.

### 2.8. Statistical analysis

All analyses were carried out using the statistical package for Social Science (v.19, IBM, Chicago). The number of positive identifications of tooth brushes were compared with that of miswaks using Chi-square test. The number of positive identifications of each oral hygiene aid were compared with immediate and after 1 month; immediate and after 2 months; immediate and after 6 months respectively. McNemer test was used for such intragroup comparisons. *P* value < .05 was considered statistically significant.

## 3. Results

**Figure 1.**
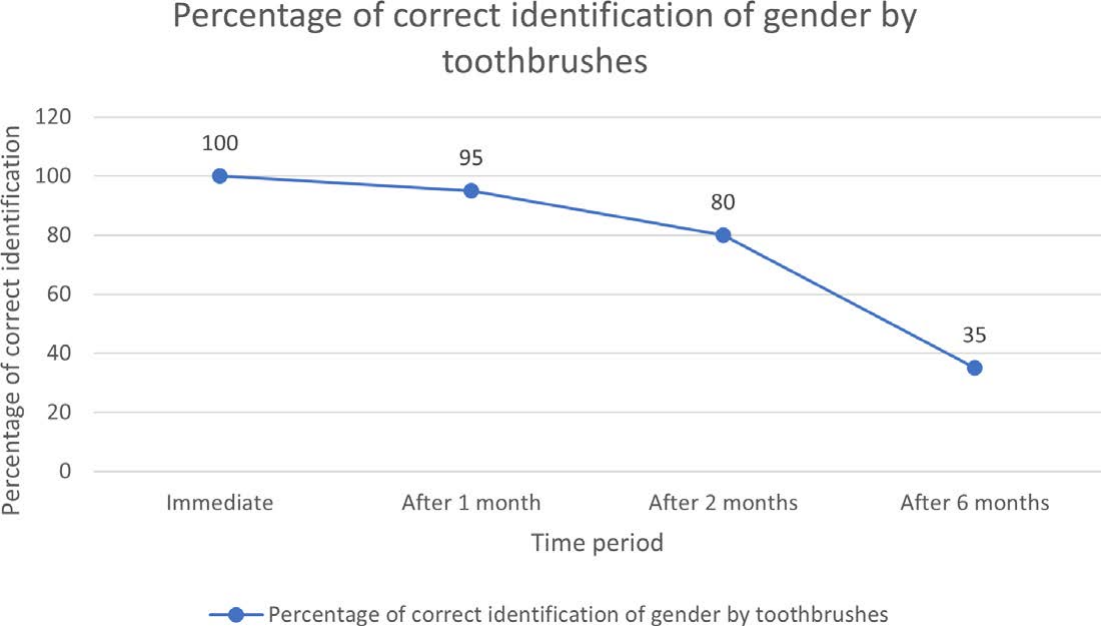
Percentage of correct identification of gender by toothbrush.

**Figure 2.**
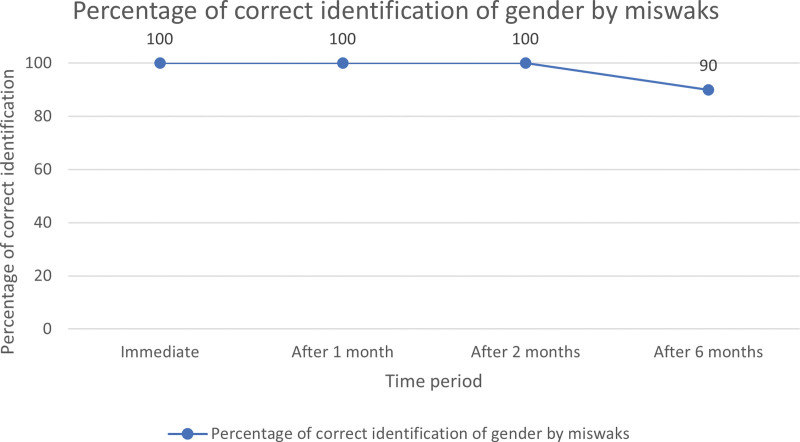
Percentage of correct identification of gender by miswaks.

Correct and positive identification of either gender was 100% when samples were obtained from toothbrush and miswak immediately. At the end of 1 month, correct and positive identification of either gender was observed in 95% samples obtained from toothbrush (Fig. 1), while it was 100% from miswak (Fig. 2), although this difference was not statistically significant. At the end of 2 months, correct and positive identification of either gender further reduced to 80% for samples obtained from toothbrushes while it was 100% for miswak. This difference was also not found to be statistically significant. At the end of 6 months, correct and positive identification of either gender was observed in 35% of toothbrush samples while it was 90% for miswak and this difference was statistically highly significant (Table 1). Intragroup comparison using McNemar test showed that the percentage difference in correct and positive identification of either gender from toothbrush samples immediately and at the end of 6 months was statistically significant (Table 2) while such a significant difference was not noted with miswak.

**Table 1:** Intergroup comparison between toothbrush and miswak as sources to collect exfoliative cells for gender identification.

Time point	Tool for collecting exfoliative cells	Identification status		*P* value
		Positive	Negative	
Immediate	Toothbrush	20 (100%)	0	-
	Miswak	20 (100%)	0	
1 mo	Toothbrush	19 (95%)	1 (5%)	.15
	Miswak	20 (100)	0	
2 mo	Toothbrush	16 (80%)	4 (20%)	.03*
	Miswak	20 (100%)	0	
6 mo	Toothbrush	7 (35%)	13 (65%)	.001*
	Miswak	18 (90%)	2 (20%)	

* *P* value < .05 - statistically significant.

**Table 2:** Intragroup comparison of toothbrush and miswak as sources to collect exfoliative cells for gender identification.

Time point	Positive identification using toothbrush	Positive identification using miswak
Immediate	20 (100%)	20 (100%)
1 mo	19 (95%)	20 (100)
2 mo	16 (80%)	20 (100%)
6 mo	7 (35%)	18 (90%)
*P* value		
Immediate vs 1 mo	.99	-
Immediate vs 2 mo	.12	-
Immediate vs 6 mo	.002*	0.5

McNemer test.

* *P* value < .05 - statistically significant.

## 4. Discussion

The present study aimed to compare the accuracy of sex determination of DNA retrieved from toothbrushes and miswaks. The accuracy was also compared between toothbrushes and miswaks at 4 different time points.

Earlier, protein markers such as ABO blood group antigens, RBC enzymes and serum proteins were used for identification of deceased individuals. However, such methods exhibited low polymorphism and showed poor stability.^[13]^ Of late, DNA markers have revolutionized forensic testing as it exhibited greater precision with higher discriminatory power and also proved to be more stable than protein markers.^[14]^ Since its advent, DNA technology has been widely accepted during crime scenes and sexual assaults as personnels involved in such crimes could be easily identified through collection of semen and blood samples. However, DNA is also subjected to degradation and many factors such as time, extreme temperature, humidity and exposure to some chemicals may hinder DNA retrieval and analysis.^[14]^ In such situations, techniques of retrieving DNA from samples other than blood specimens need to be explored. Retrieval of DNA from tooth brushes was one such alternative method used in forensics and its yield has been evaluated by previous studies.^[15]^ A recent systematic review also concluded that toothbrushes are excellent sources for DNA retrieval and sex determination.^[2]^ Recently, other sources of oral hygiene aids such as miswaks were also used in DNA retrieval.^[3]^ Considering the adequacy in availability of evidences regarding purity and yield assessment of DNA retrieved from tooth brushes and miswaks, the present study focused on evaluating and comparing the accuracy of sex determination between toothbrushes and miswaks following DNA retrieval.

According to a study conducted by Tanaka et al, 2000 the entire bundle of toothbrush bristles was able to retrieve about 430 ng/µL of DNA.^[12]^ However, in a later study conducted by Bandhya et al, 2007, it was affirmed that DNA yield and purity was better when they are retrieved from limited number of toothbrush bristles than the entire bundle.^[15]^ The entire bundle of toothbrush despite giving more genetic material, consisted of PCR inhibitors resulting from residues of toothpaste.^[16]^ Hence, it was suggested to retrieve minimum amount of DNA to run a better PCR test. This study thus used only DNA of 25 to 50 ng for each PCR test. Similar studies conducted by authors retrieved only minimal amount of DNA for PCR tests.^[17–19]^

Moreover, the chances of DNA degradation must also be considered before recommending oral hygiene aids as a successful tool in sex determination. Hence, present study explored the sex determining property of DNA retrieved from toothbrushes and miswaks at multiple time points. For this purpose, the toothbrushes and miswaks were stored at room temperature at the laboratory for 6 months to simulate real-life scenarios. A previous study conducted by Reimer compared the purity of DNA obtained from toothbrushes over 3-month period and concluded that no significant differences exist in purity of DNA collected at various time points.^[1]^ Hence, the present study was contemplated to increase the storage of samples till 6 months.

From the results, it is evident that both toothbrushes and miswaks identified the sex of participants correctly when samples were analyzed immediately after collection. However, it was interesting to note that miswaks had more positive identifications of sex than toothbrushes at the end of 6 months. Few studies have indicated the microbial contamination of toothbrushes after 3 months of storage^[20,21]^ and this could have probably contributed to less accuracy of sex determination by toothbrushes. On the other hand, miswaks due to their anti-bacterial effects would have initiated effective retrieval of DNA without contamination, thus increasing the accuracy of sex determination.^[22]^ Also, toothbrushes contain few toothpaste residues in their bristles which might hinder the purity of DNA retrieval. These tooth paste residues can potentially affect the mechanism of PCR. On contrast, miswaks are always used as fresh pieces without addition of any external agents such as toothpastes or toothpowder. Thus, being a natural product and unadulterated by chemicals, miswaks can be considered as a good source of DNA sample.

According to the study conducted by Mannakandath, the purity of DNA samples retrieved from miswaks and toothbrushes were found to be similar throughout their 5-month period of storage.^[3]^ However, a study conducted by Alfadaly showed contrast results. In their study, a complete DNA profiling was achieved in 56% of toothbrush samples and 96% of miswak samples, thus proving the superiority of miswak over toothbrush in achieving complete DNA sequencing.^[23]^ Similarly, the present study results can be probably due to the complete DNA sequencing underwent by miswak.

A previous study analyzed the sex determining region Y in the SRY gene.^[6]^ However, the present study used both SRY and ALT for identification of sex. This is mainly to record the number of samples showing positive identifications of the respective sex. The use of real-time PCR can be considered as a strength of the study as it requires less time and less-intensive labor. It reduces the chances of contamination more than the conventional PCR as it is devoid of post-amplification procedure.^[24]^ This study did not estimate the sensitivity and specificity of miswaks and toothbrushes for sex determination. This might be considered as a limitation. However, both the oral hygiene aids have been proven to have good sensitivity and specificity in sex determination.^[6]^ Hence, the present study is limited only to prove a superior oral hygiene aid for sex determination. The real-life practical difficulties are the second major limitation in recommending miswaks as better oral hygiene aid for sex determination. Tooth brushes are never interchanged as it is a unique property of an individual and can be collected from the individual house at any time. On contrast, collection of miswaks in real-life scenario might be difficult as they are used as fresh pieces and will be discarded after use. But it is prudent to note the increased accuracy of miswak for sex determination in this study. Within the above-mentioned limitations, it should be noted that miswaks when available can produce correct identification of sex even if collected several months after use.

## 5. Conclusion

From the study results, it has been concluded that miswak is a better tool to harvest exfoliated cells for gender identification when compared to a toothbrush. Hence, miswak can serve as an aid in forensic medicine for gender identification.

## Author contributions

**Conceptualization:** Abdullah Alqarni, Shaik Mohamed Shamsudeen, Master Luqman Mannakandath.

**Data curation:** Shaik Mohamed Shamsudeen, Master Luqman Mannakandath.

**Formal analysis:** Shaik Mohamed Shamsudeen, Shaik Mohammed Asif.

**Investigation:** Abdullah Alqarni, Shaik Mohamed Shamsudeen, Khalil Ibrahim Assiri.

**Resources:** Shaik Mohamed Shamsudeen, Shaik Mohammed Asif, Saeed Alassiri.

**Software:** Master Luqman Mannakandath, Shaik Mohammed Asif.

**Supervision:** Shaik Mohammed Asif, Saeed Alassiri, Khalil Ibrahim Assiri.

**Validation:** Abdullah Alqarni, Khalil Ibrahim Assiri.

**Writing – original draft:** Shaik Mohamed Shamsudeen, Master Luqman Mannakandath, Shaik Mohammed Asif.

**Writing – review & editing:** Abdullah Alqarni, Saeed Alassiri, Khalil Ibrahim Assiri.
